# Influence of Vacancy Defect on Surface Feature and Adsorption of Cs on GaN(0001) Surface

**DOI:** 10.1155/2014/490853

**Published:** 2014-07-07

**Authors:** Yanjun Ji, Yujie Du, Meishan Wang

**Affiliations:** ^1^Department of Optoelectronic Engineering, Binzhou University, Binzhou 256603, China; ^2^Institute of Electronic Engineering and Opto-Electric Technology, Nanjing University of Science and Technology, Nanjing 210094, China; ^3^School of Physics and Optoelectronic Engineering, Ludong University, Yantai 264025, China

## Abstract

The effects of Ga and N vacancy defect on the change in surface feature, work function, and characteristic of Cs adsorption on a (2 × 2) GaN(0001) surface have been investigated using density functional theory with a plane-wave ultrasoft pseudopotential method based on first-principles calculations. The covalent bonds gain strength for Ga vacancy defect, whereas they grow weak for N vacancy defect. The lower work function is achieved for Ga and N vacancy defect surfaces than intact surface. The most stable position of Cs adatom on Ga vacancy defect surface is at T_1_ site, whereas it is at B_Ga_ site on N vacancy defect surface. The *E*_ads_ of Cs on GaN(0001) vacancy defect surface increases compared with that of intact surface; this illustrates that the adsorption of Cs on intact surface is more stable.

## 1. Introduction

Due to its important characteristics such as wide band gap, high thermal conductivity, high breakdown voltage, high melting point, and chemical stability, among others, GaN and its compounds such as GaN_*x*_As_1−*x*_ [1–3] have emerged as a new type material for the fabrication of optoelectronic devices in the blue and ultraviolet spectral region [4–10]. Most research has focused on its physical properties, growth mechanisms, and surface structures [11–15]. The (0001) surface of Wurtzite GaN has good surface state and simple production process [16], the surface size of GaN(0001) is different, and the reconstruction is different in atomic structure [17, 18]. The 2 × 2 surface is considered the most stable surface [19], while a 2 × 2 Ga adatom model is thermodynamically favored under moderately Ga-rich conditions [20]. The surface stability and conductivity properties of GaN(0001) are superior to that of (000$\overset{-}{1}$) [13, 21]. GaN(0001) surface becomes the focus of attention of optoelectronics community. The effective negative electron affinity has been demonstrated for *p*-GaN(0001) surfaces after Cs adsorption or Cs and O activating [22–27]; this is important for vacuum-type optoelectronic devices.

Defects may be produced in GaN materials growth process and interact with carriers. The existence of defects will affect the adsorption of Cs on GaN surface and then affect the performances of photoelectric devices. Theoretical studies of the eigen defects of GaN have been reported [28–30]; however, the influence of such defects on the adsorption of Cs on GaN surface has not been determined. In this paper, we employ plane-wave with ultrasoft pseudopotential method to study the influence of Ga and N vacancy defects on the surface feature and adsorption of Cs on GaN(0001) surface based on the first-principle density functional theory (DFT).

## 2. Computational Methods

**Figure 1:**
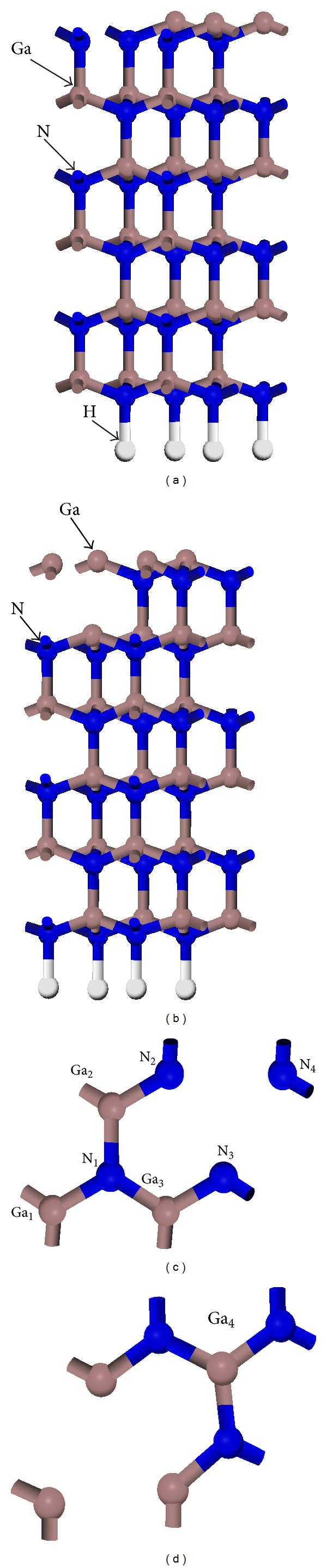
2 × 2 GaN(0001) surface: (a) side view of Ga vacancy defect surface, (b) side view of N vacancy defect surface, (c) top view of Ga vacancy defect surface, and (d) top view of N vacancy defect surface. Ga and N atoms are marked.

The parameters for the optimized bulk wurtzite GaN structures are *a* = *b* = 0.3189 nm and *c* = 0.5185 nm [31]. All calculations were performed with the quantum mechanics program Cambridge Serial Total Energy Package [32] based on density functional theory (DFT). The Broyden-Fletcher-Goldfarb-Shanno algorithm was used to relax the structure of the crystal model. The convergence precision was set to an energy change < 2 × 10^−6^ eV/atom, force < 0.005 eV/nm, convergence tolerance of a single atomic energy < 1 × 10^−5^ eV/atom, stress < 0.05 GPa, and change in displacement < 0.0001 nm in an iterative process. The surface slab was modeled with six GaN(0001) bilayers of which the lower three bilayers were fixed in the bulk configurations and a vacuum region equivalent to six GaN bilayers with overall approximate length of 1.3 nm was required. The bottom side of the slab was saturated with pseudohydrogen atoms to prevent transfer of surface charges (shown in Figure 1). Wave functions were expanded in a plane-wave basis set up to an energy cutoff of 400 eV and integrations over the Brillouin zone were performed using a 4 × 4 × 1 Monkhorst-Pack set sampling-point scheme for the surface supercell. The electron exchange and correlation were treated by using the Perdew-Burke-Ernzerhof (PBE) formulation of the generalized gradient approximation (GGA) [33].

**Figure 2:**
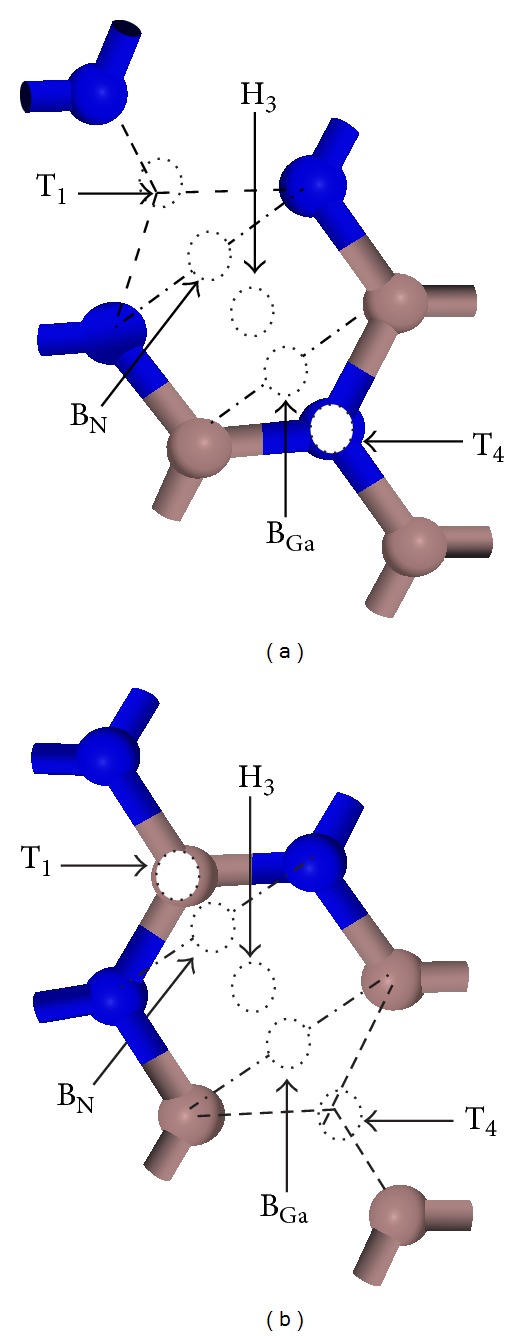
Top view of various possible adsorption sites of Cs on 2 × 2 GaN(0001) defect surface: (a) Cs on Ga defect surface, (b) Cs on N defect surface.

One Ga atom or one N atom on the outmost layer of 2 × 2 GaN(0001) surface was removed in researching vacancy defect surface, respectively (shown in Figure 1).  Figure 1(a) shows the side view of Ga vacancy defect surface and (c) is the top view.  Figure 1(b) shows the side view of N vacancy defect surface and (d) is the top view. For a Cs adsorption on GaN(0001) surface, five typical adsorption models including sites of T_1_ (Ga top), H_3_ (hollow site), T_4_ (N top), B_Ga_ (Ga bridge), and B_N_ (N bridge) were chosen [34, 35]. In this paper, these five typical adsorption sites are adopted to study the influence of Ga and N vacancy defects on the adsorption of Cs on GaN(0001) surface.  Figure 2 shows the top view of Cs at T_1_, H_3_, T_4_, B_Ga_, and B_N_ sites on GaN(0001) defect surface.  Figure 2(a) shows the top view of Cs on Ga vacancy defect surface and Figure 2(b) is Cs on N vacancy defect surface.

## 3. Deficient Surface

Based on the mean of atoms coordinates, the thickness of the first bilayer is calculated; it is 0.0272 nm for Ga vacancy defect surface, whereas it is 0.0436 nm for N vacancy defect surface and 0.0653 nm for intact surface, compared with an ideal value 0.0647 nm. It has the biggest change in surface feature for Ga vacancy defect surface after relaxation. To study the reason for the change of surface feature, the charges of atoms in the outmost layer are shown in Table 1; the overlap populations and bond lengths between Ga and N atoms in the outmost layer of intact, Ga vacancy defect, and N vacancy defect surfaces are shown in Table 2.

The overlap population increases, the bond length decreases, and the covalent bond between Ga and N gains strength for Ga vacancy defect surface as shown in Table 2 caused mainly by the increase of charges of every Ga atom (see Table 1) compared with that of intact surface.

The overlap population decreases, covalent bond is weakened, and bond length between Ga and N increases for N vacancy defect surface as shown in Table 2 caused mainly by the decrease of charges of N and Ga atoms (see Table 1) compared with that of intact surface.

The main reason for the change of surface feature is the break of atoms bonds in the outmost surface layer, there are uncompensated electrons, and dipole moment directing to outside is formed. Compared with the intact surface, the charges of every Ga atom in Ga vacancy defect surface increases, while the total charges of Ga atoms in the outmost layer decrease, so the dipole moment increases. The thickness of the first bilayer is compacted by means of strong dipole moment and covalent bond. There is a preponderance of uncompensated electrons for N vacancy defect surface; the dipole moment is the max, but the change in the first layer thickness is less than that of Ga vacancy defect surface by means of the weak covalent bond.

For semiconductors, the work function is the minimum energy needed by electrons at the bottom of the semiconductor to escape externally. The easier the escape is, the larger dipole moment directing to outside is formed, the lower work function is. The size of the dipole moment among three surfaces is intact surface < Ga vacancy defect surface < N vacancy defect surface. Work functions of three surfaces are 4.20 eV (in agreement with Re.18), 4.054 eV, and 4.052 eV, respectively (shown in Table 3). Calculations show that the defect favors the escape of electrons but can induce bigger change in surface feature.

**Figure 3:**
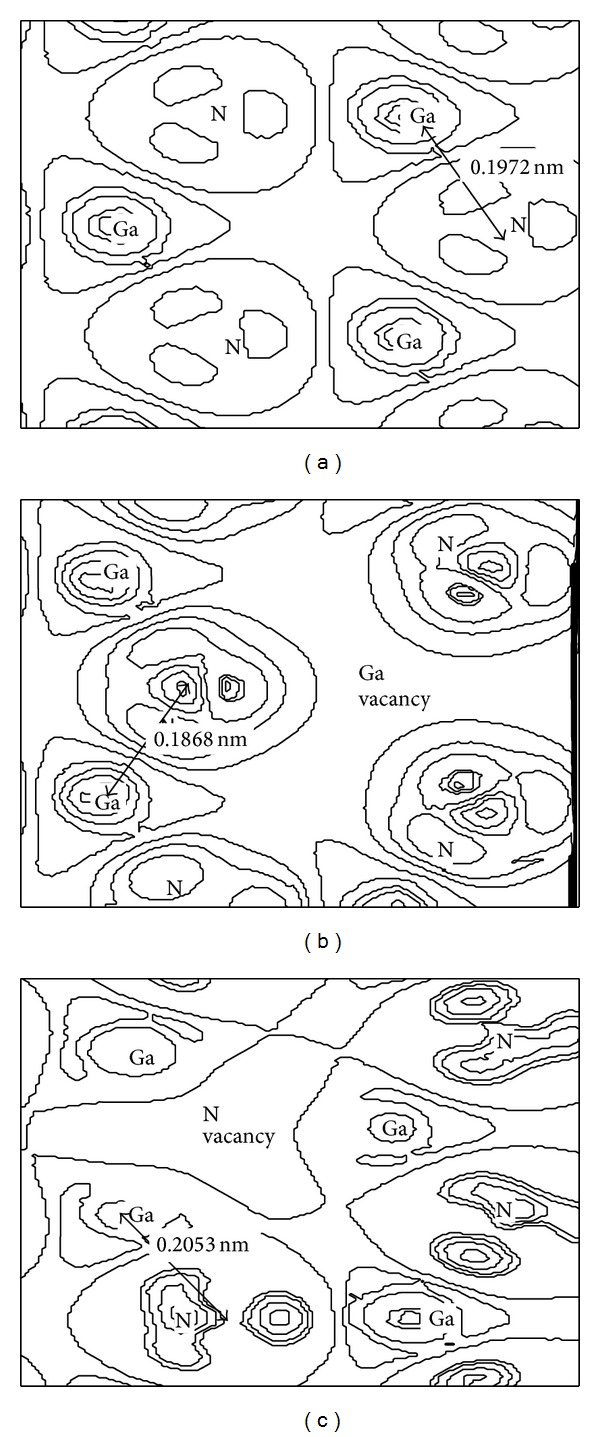
Electron density difference in the (0001) plane of the surface atoms: (a) intact surface, (b) Ga vacancy defect surface, and (c) N vacancy defect surface.

The charge density difference in the (0001) plane of the surface atoms is shown in Figure 3 to indicate the influence of Ga, N defect on the interaction between atoms visually, in which the bond length is also given. The electron cloud of the N atoms is uniformly distributed in three directions, joining to Ga atoms in the (0001) plane for intact surface, as shown in Figure 3(a). The degree of electron aggregation enlarges after Ga defect (Figure 3(b)), the overlap population increases, the covalent bond of Ga-N is stronger and the bond length of Ga-N is shorter than that of intact surface. The overlap population between the Ga and N atoms gets its minimum after N defect (Figure 3(b)); the bond length of Ga-N increases.

## 4. Cs Adsorption on GaN(0001) Deficient Surface

To study the effect of defect on the adsorption of Cs on GaN(0001) surface, first, a Cs was placed at H_3_ site on Ga and N vacancy defect surfaces above surface 0.1 nm moving freely in relaxation process and, then, was fixed at five high symmetry sites moving only in *Z* direction.

Adsorption energy *E*_ads_ was calculated as the difference between the total energy of the GaN(0001) slab with adsorbed Cs and the sum of the total energies of the clean surface and isolated Cs atom [25]. The *E*_ads_ of a Cs at these six sites on Ga vacancy defect surface is shown in Table 3. The results show that *E*_ads_ is negative no matter Cs is at the five high symmetry sites or moves freely; it means that the absorption process is an exothermic chemical process and is stable. The final convergence position of Cs is above the Ga vacancy when it moves freely on Ga vacancy defect surface. The *E*_ads_ of every symmetry site decreases compared with that of Cs on intact surface. The most stable adsorption site is T_1_ site (top of Ga vacancy), no longer the B_N_ and H_3_ sites for intact surface [26]; the most unstable adsorption site is T_4_ site. The lack of Ga atom makes the repulsive interaction of Ga to Cs nonexistent and Cs be attracted only by three N atoms, so the stable adsorption site changes compared with that of intact surface. Since the increase of uncomplexed electrons after Ga absence leads to the increasing of dipole moment; work functions of Ga vacancy defect surface with Cs at different high symmetry sites decline slightly compared with that of intact surface.

The *E*_ads_ is negative no matter a Cs is at the five high symmetry sites or moves freely on N vacancy defect surface (shown in Table 3); it means that the absorption process is also stable. The results show that *E*_ads_ of every symmetry site decreases compared with that of Cs on intact surface, while it is bigger than that of Cs on Ga vacancy defect surface. B_Ga_ is the most stable adsorption site in agreement with the case when Cs moves freely on N vacancy defect surface; H_3_ is not convergence different from the case of intact surface. Since the increase of uncomplexed electrons after N absence leads to the increasing of dipole moment, work functions of N vacancy defect surface in different high symmetry sites decline slightly compared with that of intact surface.

Comparing the *E*_ads_ of Cs on Ga and N vacancy defect surfaces, it can be seen that the adsorption of Cs on N vacancy defect surface is more stable than on Ga vacancy defect surface. The lower work function is achieved for Ga and N vacancy defects surfaces than intact surface in agreement with the analysis of dipole moments before.

## 5. Discussion and Conclusions

The change of surface feature has been compared between GaN(0001) vacancy defect and intact surfaces. Adsorption characteristic and change in work function of a Cs atom on GaN(0001) (2 × 2) vacancy defect surface have been investigated using DFT with a plane-wave ultrasoft pseudopotential method based on first-principles calculations, compared with that of intact surface. Results show that Ga and N vacancy defects may cause contraction of the first bilayer and decrease of surface work function for the increase of dipole moment. The most stable adsorption site is T_1_ site (top of Ga vacancy) for Ga vacancy defect surface, while it is B_Ga_ site for N vacancy defect surface. When the Cs was adsorbed on the vacancy defect surfaces, work functions for different high symmetry sites decline slightly compared with that of intact surface, but *E*_ads_ increases, this illustrates that the adsorption of Cs on intact surface is more stable.

## Figures and Tables

**Table 1:** Charges of atoms in the outmost layer of intact, Ga vacancy defect, and N vacancy defect surfaces.

Surface	Atoms and its charges/e							
	Ga_1_	Ga_2_	Ga_3_	Ga_4_	N_1_	N_2_	N_3_	N_4_
Intact	0.75	0.75	0.75	0.75	−0.98	−0.98	−0.98	−0.98
Ga vacancy defect		0.88	0.88	0.88	−1.04	−0.97	−0.98	−0.97
N vacancy defect	0.79	0.35	0.35	0.27		−0.94	−0.94	−0.95

**Table 2:** N–Ga bond lengths and overlap populations of intact, Ga vacancy defect, and N vacancy defect surfaces.

Bonds	Intact surface		Ga vacancy defect surface		N vacancy defect surface		
	Overlap population	Bond length/nm	Overlap population	Bond length/nm	Bonds	Overlap population	Bond length/nm
N_1_–Ga_1_	0.56	0.1974	0.73	0.1868	N_2_–Ga_4_	0.47	0.2053
N_1_–Ga_2_	0.55	0.1972	0.73	0.1868	N_3_–Ga_4_	0.47	0.2053
N_1_–Ga_3_	0.58	0.1967	0.65	0.1970	N_4_–Ga_4_	0.53	0.2012

**Table 3:** *E*_ads_ and work functions of Cs on GaN (0001) intact, Ga vacancy defect, and N vacancy defect surfaces.

Adsorption site	Intact surface		Ga vacancy defect		N vacancy defect	
	*E*_ads_/eV	Work function/eV	*E*_ads_/eV	Work function/eV	*E*_ads_/eV	Work function/eV
H_3_	−2.02	2.16	−1.15	2.46		Misconvergence
T_1_	−1.89	2.30	−1.89	2.28	−1.55	2.30
T_4_	−1.96	2.37	−0.80	2.28	−1.48	2.23
B_Ga_	−1.98	2.36	−0.84	2.22	−1.57	2.20
B_N_	−2.04	2.36	−0.84	2.33	−1.54	2.12
Freedom	−2.04	2.42	−1.99	2.67	−1.53	2.19

## References

[1] Reshak AH (2014). Dispersion of the second harmonic generation in GaN_*x*_ As_1-*x*_(*x* = 0.25, 0.5, 0.75) alloys. *Journal of Alloys and Compounds*.

[2] Reshak AH, Charifi Z, Baaziz H (2013). The influence of the lattice relaxation on the optical properties of GaN_x_As_1-x_ alloys. *Solar Energy*.

[3] Baaziz H, Charifi Z, Reshak AH, Hamad B, Al-Douri Y (2012). Structural and electronic properties of GaN_x_ As_1-x_ alloys. *Applied Physics A*.

[4] Hass Bar-Ilan A, Zamir S, Katz O, Meyler B, Salzman J (2001). GaN layer growth optimization for high power devices. *Materials Science and Engineering A*.

[5] Zhao H, Liu G, Tansu N (2010). Analysis of InGaN-delta-InN quantum wells for light-emitting diodes. *Applied Physics Letters*.

[6] Chow WW (2011). Modeling excitation-dependent bandstructure effects on InGaN light-emitting diode efficiency. *Optics Express*.

[7] Farrell RM, Hsu PS, Haeger DA (2010). Low-threshold-current-density AlGaN-cladding-free m -plane InGaN/GaN laser diodes. *Applied Physics Letters*.

[8] Zhang J, Zhao H-P, Tansu N (2011). Large optical gain AlGaN-delta-GaN quantum wells laser active regions in mid- and deep-ultraviolet spectral regimes. *Applied Physics Letters*.

[9] Wang XH, Chang BK, Du YJ, Qiao JL (2011). Quantum efficiency of GaN photocathode under different illumination. *Applied Physics Letters*.

[10] Wang XH, Chang BK, Ren L, Gao P (2011). Influence of the *p*-type doping concentration on reflection-mode GaN photocathode. *Applied Physics Letters*.

[11] Kapolnek D, Keller S, Vetury R (1997). Anisotropic epitaxial lateral growth in GaN selective area epitaxy. *Applied Physics Letters*.

[12] Brandt MS, Herbst P, Angerer H, Ambacher O, Stutzmann M (1998). Thermopower investigation of *n*- and *p*-type GaN. *Physical Review B*.

[13] Motohashi K, Hosoya K, Imano M, Tsurubuchi S, Koukitu A (2007). Analyses of GaN (0 0 0 1) and $\left(\text{000}\overset{-}{1}\right)$ surfaces by highly-charged ions. *Surface Science*.

[14] Du Y, Chang B, Fu X, Wang X, Wang M (2012). Electronic structure and optical properties of zinc-blende GaN. *Optik*.

[15] Timon V, Brand S, Clark SJ, Gibson MC, Abram RA (2005). First-principles calculations of 2 × 2 reconstructions of GaN(0001) surfaces involving N, Al, Ga, In, and As atoms. *Physical Review B: Condensed Matter and Materials Physics*.

[16] Nakamura S, Fasol G (1997). *The Blue Laser Diode*.

[17] Rosa AL, Neugebauer J (2006). First-principles calculations of the structural and electronic properties of clean GaN (0001) surfaces. *Physical Review B. Condensed Matter and Materials Physics*.

[18] Hattori AN, Endo K, Hattori K, Daimon H (2010). Surface treatments toward obtaining clean GaN(0 0 0 1) from commercial hydride vapor phase epitaxy and metal-organic chemical vapor deposition substrates in ultrahigh vacuum. *Applied Surface Science*.

[19] Rapcewicz K, Nardelli MB, Bernholc J (1997). Theory of surface morphology of wurtzite GaN (0001) surfaces. *Physical Review B. Condensed Matter and Materials Physics*.

[20] Feenstra RM, Northrup JE, Neugebauer J (2002). Review of structure of bare and adsorbate-covered GaN(0001) surfaces. *MRS Internet Journal of Nitride Semiconductor Research*.

[21] Du Y, Chang B, Zhang J, Li B, Wang X (2012). First-principles study of the electronic structure and optical properties of GaN(0001) surface. *Acta Physica Sinica*.

[22] Pakhnevich AA, Bakin VV, Yaz'kov AV (2004). Energy distributions of photoelectrons emitted from p-GaN(Cs, O) with effective negative electron affinity. *JETP Letters*.

[23] Machuca F, Liu Z, Maldonado JR, Coyle ST, Pianetta P, Pease RFW (2004). Negative electron affinity group III-nitride photocathode demonstrated as a high performance electron source. *Journal of Vacuum Science and Technology B: Microelectronics and Nanometer Structures*.

[24] Qiao J, Tian S, Chang BK, Du X, Gao P (2009). Progress in study of negative electron affinity GaN vacuum surface electron source. *Chinese Physical Society*.

[25] Du X, Chang B, Qian Y, Fu R, Gao P, Qiao J (2010). Activation technique of GaN negative electron affinity photocathode. *Chinese Journal of Lasers*.

[26] Du Y, Chang B, Wang X, Zhang J, Li B, Wang M (2012). Theoretical study of Cs adsorption on GaN(0 0 0 1) surface. *Applied Surface Science*.

[27] Shen Y, Kang J (2002). Ab initio calculation of the electronic structure of carbon and oxygen impurities in GaN. *Acta Physica Sinica*.

[28] Pang C, Shi J, Zhang Y (2007). Electronic structures of wurtzite GaN with Ga and N vacancies. *Chinese Physics Letters*.

[29] Jie WW, Yang C (2010). The electronic structures of vacancy defects in hexagonal GaN. *Journal of Sichuan Normal University (Natural Science)*.

[30] Du Y, Chang B, Wang H, Zhang J, Wang M (2012). First principle study of the influence of vacancy defects on optical properties of GaN. *Chinese Optics Letters*.

[31] Perlin P, Jauberthie-Carillon C, Itie JP, San Miguel A, Grzegory I, Polian A (1992). Raman scattering and x-ray-absorption spectroscopy in gallium nitride under high pressure. *Physical Review B*.

[32] Segall MD, Lindan PJD, Probert MJ (2002). First-principles simulation: ideas, illustrations and the CASTEP code. *Journal of Physics Condensed Matter*.

[33] Perdew JP, Burke K, Ernzerhof M (1996). Generalized gradient approximation made simple. *Physical Review Letters*.

[34] Hu C, Chen Y, Li J, Zhang Y (2008). First-principles calculations of ethanethiol adsorption and decomposition on GaN (0 0 0 1) surface. *Applied Surface Science*.

[35] Sun Q, Selloni A, Myers TH, Doolittle WA (2006). Oxygen adsorption and incorporation at irradiated GaN(0001) and GaN(000 1*®*) surfaces: first-principles density-functional calculations. *Physical Review B. Condensed Matter and Materials Physics*.

